# Ethical Dimensions of Human Immunodeficiency Virus Infection During Pregnancy

**DOI:** 10.1155/S106474499700029X

**Published:** 1997

**Authors:** Frank A. Chervenak, Laurence B. McCullough, William J. Ledger

**Affiliations:** 1 Department of Obstetrics and Gynecology New York Hospital-Cornell Medical Center 525 East 68th Street, J130 New York NY 10021 USA; 2 Center for Medical Ethics and Health Policy Baylor College of Medicine Houston TX USA

## Abstract

Physicians encounter complex and sensitive ethical challenges in the medical care of pregnant women with human immunodeficiency virus (HIV) infection. This paper identifies those ethical challenges and provides concrete clinical guidance for how they should be addressed in obstetric care. The paper begins with a brief historical review, to highlight and to call into question the civil rights model of the ethics of HIV infection that has dominated the literature, clinical practice, and public policy. The authors propose an alternative ethical framework. This framework begins by underscoring the public health obligations of both physicians and pregnant women with HIV infection. The framework is based on a clinical ethics that appeals to both beneficence-based and autonomy-based obligations of the physician to the pregnant woman and the beneficence-based obligations of both the physician and the pregnant woman to the fetal patient. This framework is then deployed in a clinical ethical analysis of termination of pregnancy and contraception, partner notification, disclosure and confidentiality of her serostatus by the patient to the health care team, disclosure and confidentiality of her serostatus to other health care professionals, prevention of vertical transmission, and advance directives.


Infectious Diseases in Obstetrics and Gynecology 5:192–198 (1997)
© 1997 Wiley-Liss, Inc.

### Ethical Dimensions of Human Immunodeficiency Virus Infection During Pregnancy

**Frank A. Chervenak,[1]* Laurence B. McCullough,[2] and William J. Ledger[1]**
[1]*Department of Obstetrics and Gynecology, New York Hospital-Cornell Medical Center, New York, NY*
[2]*Center for Medical Ethics and Health Policy, Baylor College of Medicine, Houston, TX*

### Abstract

Physicians encounter complex and sensitive ethical challenges in the medical care of pregnant women with human immunodeficiency virus (HIV) infection. This paper identifies those ethical challenges and provides concrete clinical guidance for how they should be addressed in obstetric care. The paper begins with a brief historical review, to highlight and to call into question the civil rights model of the ethics of HIV infection that has dominated the literature, clinical practice, and public policy. The authors propose an alternative ethical framework. This framework begins by underscoring the public health obligations of both physicians and pregnant women with HIV infection. The framework is based on a clinical ethics that appeals to both beneficence-based and autonomy-based obligations of the physician to the pregnant woman and the beneficence-based obligations of both the physician and the pregnant woman to the fetal patient. This framework is then deployed in a clinical ethical analysis of termination of pregnancy and contraception, partner notification, disclosure and confidentiality of her serostatus by the patient to the health care team, disclosure and confidentiality of her serostatus to other health care professionals, prevention of vertical transmission, and advance directives. Infect. Dis. Obstet. Gynecol. 5:192–198, 1997.
© 1997 Wiley-Liss, Inc.

Key words
HIV infection; pregnancy; ethics; confidentiality; prevention; advance directives

When human immunodeficiency virus (HIV) and HIV-related infections were first described in the medical literature in the United States early in the last decade, several factors shaped the societal response to persons with HIV and those thought to be at risk for infecting others with HIV. First, developed countries, such as the United States, at that time had assumed that microbes had been defeated and that the age of infectious diseases had passed. This was such a common view in the lay and medical communities that in the late 1970s research funds for infectious disease had become constrained. Medicine, everyone seemed to assume, had tamed microbes, bringing nature under human control. Infectious diseases, especially life-threatening infectious diseases, did not affect countries like the United States; these diseases were a third-world problem. Thus, the pandemic of HIV came as a shock to a society that, it turned out in retrospect, had deluded itself about the persistence of microbes and their role in disease. As a consequence, our response to HIV was anything but calm, thoughtful, and balanced.

Second, living experience with life-threatening

*Correspondence to: Dr. Frank A. Chervenak, Department of Obstetrics and Gynecology, New York Hospital-Cornell Medical Center, 525 East 68th Street, J130, New York, NY 10021.

Received 1 October 1997

Accepted 21 October 1997

occupational infectious diseases was confined to older physicians, who had trained during the era before widespread use of antibiotics. These physicians had experienced, and many of them had accepted, the ethical challenge of accepting risk to themselves as part of medical training and the care of patients; however, most of their younger colleagues had not. As a consequence, the response of physicians themselves to the first reports of HIV lacked the intellectual, moral, and emotional discipline that should mark the professional person.[1] Perhaps because they had been taught that microbes were in retreat, physicians thought that they should not have to expect, much less take, risks to their own health and lives in the care of patients. At the very time at which society should have been able to look to physicians as role models for a calm, intellectually disciplined response to life-threatening infections, many physicians let society down. Everyone remembers the avoidance behavior that was employed, as well as some of the more unusual responses to possible exposure, e.g., surreptitious prophylactic use of drugs such as azidothymidine (AZT) and gowning and gloving to conduct psychiatric interviews.

Third, at-risk populations in the United States in the 1980s included mainly those who shared needles during intravenous (IV) drug use and those who engaged in male homosexual sexual intercourse. The first group surely was politically marginalized in our society. As a consequence, the ability to marshall responses of sympathy, much less justice, became very limited. The harsh reality is that this population was written off in public policy. The second group, homosexually active men, confronted HIV infection at a time in the political history of our country when the homosexual community had made considerable political advances and was determined not to lose what they had struggled to gain. This posture reflected the history of minority groups struggling for recognition, equality, and political power in our country. Moreover, in key cities, such as New York, San Francisco, and Houston, as well as key states, such as California and New York, homosexuals represented a political constituency with which elected officials—and those seeking elected office—had to reckon.

The result of these and other factors that historians will one day sort out more thoroughly was that a civil rights model dominated the ethical and public policy debates about HIV infection. The issues concerned lack of access to health care by an already discriminated-against, though increasingly politically powerful minority, preventing and punishing employment discrimination, and preventing the very real possibility that all of the progress since the Stonewall uprising in New York City in 1969 would be lost if HIV were treated as what in fact it was and remains, an infectious disease-public health problem. It should have come as no surprise that the rights of those with HIV became of paramount importance, eclipsing such relevant ethical considerations as the obligation of those with dangerous infectious diseases and of health care professionals to prevent such diseases being transmitted to others. In this rights-dominated climate of opinion, it also should have come as no surprise that the enforcement of such an obligation by health care professionals and public health law did not become a viable option.

Things have changed since the early 1980s. There now exist powerful legal protections for those with HIV, particularly the Americans with Disabilities Act.[2,3] We have now had enough experience with HIV that it is increasingly possible to rethink the clinical and public health ethics of HIV infection. This change has been accelerated by the advance of new therapeutic regimens that promise to transform HIV infection from a lethal to a chronic health problem. The exclusive emphasis on the rights of those with HIV infection to the exclusion of their ethical obligations to others and the enforcement of those obligations by clinicians and public health officials should, we believe, now give way to a more ethically balanced approach. With such an approach there are indeed substantial rights-based obligations to those with HIV infection, principally to see to it that they have reliable access to diagnostic services and continuity of care.[4] There are also obligations of such individuals to protect others from preventable transmission of HIV, especially when those others are incapable of consenting to such exposure.

### CLINICAL ETHICAL FRAMEWORK FOR THE MANAGEMENT OF HIV INFECTION OF PREGNANT WOMEN AND NEWBORNS

These considerations set the stage for the development of a clinical ethical framework for the management of HIV-infected pregnant women and newborns. First, we identify an ethical framework for the public health dimensions of care for these two populations. Second, we develop an ethical framework for the clinical ethical issues that arise in the care of these patients.

### Public Health Ethics for HIV Infection

The ethics of public health management of these patients begins with securing their right to reliable and adequate clinical management of their problems.[4] Given that the United States does not have a centrally organized health care system, meeting the requirements of such a right falls to state and local government, institutions, and health care professionals. We urge physicians to be in the forefront of advocacy for the funds required to meet the clinical needs of these patients. This advocacy should be directed at government, institutions, and oneself and one's colleagues. Many pregnant women with HIV and their infected newborns come from the lower socioeconomic groups in our society.[4] Because they are often politically marginalized, physicians and health care institutions must advocate further, if only because no one else appears willing to do so. This leads to an important ethical principle of the public health ethics of HIV infection—justice. In the present context, justice involves the obligation to meet the needs of those who are among the least well off and most vulnerable members of our society.

### Clinical Ethics for HIV Infection

The clinical ethics of HIV infection involves two well-known principles of clinical ethics. The first of these ethical principles is beneficence, which obligates the physician to seek for the patient a greater balance of goods over harms as those goods and harms are understood and balanced from a rigorous clinical perspective.[1] In the case of HIV infection, beneficence creates a cascade of obligations: to prevent the transmission of HIV when there are safe and effective means for doing so; to undertake vigorous measures of secondary and tertiary prevention for patients with HIV infection; and to meet the needs of patients dying from end-stage HIV infections. The physician has inescapable beneficence-based obligations to newborns, as well as to pregnant women and, in a pregnancy being taken to term, to the fetal patient. Pregnant women also have parallel beneficence-based obligations to the fetal patient and the future newborn that it will become.[1]

The second ethical principle of the clinical ethics of HIV infection is respect for autonomy. This principle is required because, in addition to a rigorous clinical perspective on any patient's interests, adult patients have their own perspective on their own interests. This perspective must be respected and taken into account by the physician. Thus, the principle of respect for autonomy obliges the physician to seek for the patient a greater balance of goods over harms for the patient, as those goods and harms are understood and balanced by the patient.[1]

The autonomy of the pregnant woman when the pregnancy is going to term is not absolute, i.e., without any exceptions or limitations. Because such pregnancies involve a fetal patient, the woman has beneficence-based obligations to the fetal patient and the newborn and child that it will become. Thus, the autonomy of a pregnant woman whose pregnancy involves a fetal patient is already limited by such beneficence-based obligations. Her primary moral relationship to her physicians, therefore, is not that of a rights bearer, as the civil rights model of HIV would have it. This model applies, if at all, only to non-pregnant individuals. Instead, the pregnant woman is both a rights *and* obligations bearer. This makes for a more complicated and nuanced moral relationship of physician and patient.

The physician caring for an HIV-infected pregnant woman when the pregnancy is going to term therefore must manage a complex set of ethical obligations: 1) beneficence-based obligations to the pregnant woman (the three described above); 2) beneficence-based obligations to the fetal patient (the same three described above); and 3) autonomy-based obligations to the pregnant woman. The woman's autonomy and therefore the physician's autonomy-based obligations to her are limited by her beneficence-based obligations to the fetal patient, which are precisely the same as the physician's three beneficence-based obligations. Whether and when the physician should in clinical practice invoke and, if necessary, enforce the pregnant woman's beneficence-based obligations becomes a central question in the clinical ethics of HIV infection.

We turn now to the clinical application of this framework. We consider ethical issues that arise in the management of HIV-infected pregnant women.

### ETHICAL ISSUES THAT ARISE IN THE MANAGEMENT OF THE HIV-INFECTED PREGNANT WOMAN

### Termination of Pregnancy and Contraception

The authors have argued elsewhere that the decision to terminate a pregnancy before viability is a function of the pregnant woman's autonomy,[1] a view held commonly in the literature on the ethics of abortion. It follows, as a matter of strict ethical obligation, that counseling the pregnant woman about the alternative of abortion should be non-directive. The physician's own personal views—whether to end the pregnancy because an HIV-infected child will have serious diseases followed by early death or to continue the pregnancy out of pro-life or other personal convictions—should not be allowed to influence the counseling process in any conscious fashion. The physician should also discipline himself or herself to minimize subtle, unconscious bias in the counseling process.

During pregnancy the options of contraception and sterilization for future pregnancies should be offered to the pregnant woman. Counseling about these matters should be non-directive. A key dimension of this counseling is providing unbiased information about the patient's present life expectancy, rates of vertical transmission, reduction of those rates by zidovudine (ZDV), the course and premature mortality resulting from HIV infection in newborns, and the psychosocial dimensions of rearing a possibly infected child when the woman herself is infected and will become ill.[5] We believe that it is possible for the conscientious physician to raise these ethically relevant matters in a sensitive and honest fashion and to help the woman, in non-directive counseling, to reflect on them carefully and thus reach an informed decision about them.

### Partner Notification

A pregnant woman who is HIV infected has the same beneficence-based obligations to others that anyone with a serious infectious disease has, obligations that have become obscured in the civil rights model of the ethics of HIV infection. Her obligations include engaging in primary prevention of the transmission of the disease to immediate partners. This is especially urgent, because her sexual partners and needle sharers may engage in these behaviors with others, further spreading the disease before they themselves learn that they are infected. Thus, anyone with HIV infection has both individual and public health obligations to prevent transmission of HIV.

These beneficence-based obligations of the pregnant woman are buttressed by her autonomy-based obligations to prevent HIV infection. Consider the following line of reasoning. Part of the mechanism of transmission of HIV involves the decisions of individuals to engage in behaviors that are known to transmit HIV. Moreover, despite widespread educational efforts, not everyone engaging in needle sharing or sexual intercourse understands HIV and its modes of transmission. Thus, those with HIV infection cannot assume that those with whom they share needles or have sexual intercourse already know and have consented to the health risks of such behaviors vis-à-vis HIV infection. Such consent must be explicit, a requirement of the ethical principle of respect for autonomy that governs all human behavior.

These beneficence-based and autonomy-based analyses combine to create a very strong ethical obligation on the part of anyone with HIV infection to notify sexual partners and needle sharers of that status. Pregnant women are no exception. Moreover, these patients may sometimes require or welcome an offer to assist in the process of notification and the physician should make such an offer as a matter of routine. If the patient is unwilling to notify partners or sharers, then the reasons for this should be elicited and explored. It may be that the woman has legitimate concerns about her own well-being and safety, e.g., from a possibly violent response.[6] Such women should first be assisted to get out of these relationships—social workers can be of great assistance in this matter—and then their partners can be notified separately.

When the patient refuses to notify others when she is placing them at risk without consent and does not have convincing reasons for doing so, the physician should make another rigorous effort to persuade the patient, by pointing out that this really is a matter of serious, urgent obligation and that she therefore owes it to her sexual partners or needle sharers to notify them. If she agrees to do so, the physician should consider a follow-up mechanism, e.g., calling the partner within a very limited time period, e.g., 24 h. If she does not agree to do so, the physician has a public health obligation and an individual beneficence-based obligation to notify partners. There has always existed a physician privilege to violate confidentiality when there is sufficient reason to do so,[7] and protecting innocent others from a serious, ultimately life-taking infection surely counts as such a reason. Some states permit such disclosure to the patient's spouse. The reader should familiarize himself or herself with relevant state law and be willing to work to change laws that prohibit disclosure. These laws reflect an excessive emphasis on the civil rights approach to HIV infection.

### Disclosure by Patient to Team

It is surely in the interest of every patient that his or her providers have a complete medical and social history. This is all the more the case for HIV infection, because HIV infection changes the patient's risk profile and response to medical interventions. Moreover, new treatments offer the promise of extended life with a chronic, not immediately fatal, illness. These matters should be explained to all patients, so that HIV-infected patients will not feel singled out for different management.

Patients with HIV infection should be counseled about their obligations regarding notification of partners and sharers, as discussed above. A clinic's or institution's policies about sexual partner and needle sharer notification should also be disclosed.

Patients should be assured that information that they provide to their health professionals is confidential information but that there are sometimes ethical and legal limits on that confidentiality. In addition to partner notification, information usually must be provided on third-party payment forms. These third parties also have ethical and legal obligations to protect the confidentiality of patients' information.

Patients who are concerned about the effect of their employer learning about HIV status should be counseled that the Americans With Disabilities Act provides them with considerable legal protection.[2,3] Every clinic or physician providing care to HIV-infected patients should have information available to patients about where to go for legal advice and counseling. This will help to assure patients who may be reluctant to disclose their serostatus for fear of job discrimination.

Patients should also be able to be confident that their serostatus will have no adverse effects on the care that they will receive or behavior of those who care for them. Actual practice and policy should be such as to make this confidence warranted. It is therefore the responsibility of every institution to make sure that policies and procedures indeed support this assumption on each patient's part.

### Disclosure to Other Health Care Professionals

Every health care professional has the same obligations of confidentiality toward patients. This applies to the primary care providers and to all consultants, including consultants who may not see the patient, e.g., in informal or "curbside" consultation. Again, patients should be able to assume this, and institutional policies and practices should assure that confidentiality is appropriately maintained.

An especially important consideration is communication between the primary physician and dentists, because of the oral manifestations of HIV infection, and between the primary care physician and mental health professionals, because of the significant rates of dementing disorders associated with HIV infection. Some state laws appear to restrict such communication, because it is mistakenly thought to violate confidentiality. Physicians should work to change such laws, because they mistakenly assume that some health professionals have different obligations of confidentiality than do others; this assumption is, quite simply, false and false assumptions do not make for sound public policy.

### Prevention of Vertical Transmission

Clinical trials of ZDV for vertical transmission of HIV have produced the impressive conclusion that the rate of vertical transmission can be reduced by as much as 75%.[8] The pregnant woman has a beneficence-based obligation to accept medical interventions that produce significant benefit for the fetal patient and child it will become when such interventions are on balance either not significantly harmful to her or may even benefit her. Studies of ZDV have raised questions about the efficacy of

ZDV on changing the course of HIV infection.[9] If clinical judgment follows such trial results, then, at worst, ZDV is of manageable risk to the pregnant woman and of very significant benefit to the fetal patient and child it will become. In a pregnancy going to term, therefore, the pregnant woman is ethically obligated to accept this preventive measure.

It follows from this that as a matter of strict ethical obligation, every pregnant woman should be offered HIV testing and ZDV should be available to every pregnant woman found to be HIV infected. If the second condition is not met, offering testing is meaningless in terms of preventing vertical transmission. Thus, policy matters directly affect clinical ethics, adding yet another advocacy obligation to the physician's agenda.

The physician's response to women who refuse to be tested for HIV should be repeated and rigorous efforts aimed at the woman's reconsidering and changing her decision on the assumption that the woman wants a good outcome for her baby. The physician should underscore the woman's obligation to prevent vertical transmission and the importance to her own care of documented serostatus.

Pregnant women taking their pregnancies to term who refuse ZDV should be vigorously persuaded to change their decisions. Counseling should be directive and vigorous. The physician should revisit the issue throughout pregnancy, since ZDV may have its preventive effect in the birth canal rather than earlier in pregnancy. Family members may be enlisted to help the pregnant woman think matters through and to help to persuade her of the wisdom of this course. Directive counseling rejects the ethical assumption that the pregnant woman has a right to refuse ZDV; rather, directive counseling is justified on the assumption that she has a beneficence-based obligation to accept it and that her freedom to refuse is therefore restricted by this obligation.

### Advance Directives

The final clinical ethical concern involves advance directives for pregnant women.[1] Every state but two now provides for advance directives in the form of living wills. A living will allows a patient to refuse life-prolonging treatment when two conditions are satisfied: the patient is terminally ill as defined by relevant statute and the patient has lost the capacity to make decisions, in reasonable medical judgment (court review is not required). All states by statute or common law recognize durable power of attorney for health care. This allows a patient to assign decision making authority to someone else, a transfer that takes effect when one condition is satisfied: the patient has lost decision making capacity in reasonable medical judgment (court review is not required). A diagnosis of terminal illness is not required.

As soon as possible after a diagnosis of HIV has been discussed with the patient, the physician should inform the patient about advance directives to underscore their importance, and urge the patient to consider completing both the living will and the durable power of attorney for health care. Patients who are reluctant to do so should be sensitively informed about two significant risks of not having such legal documents. First, decisions might be made about the patient's management that would differ from those the patient would want. Second, individuals might be asked to make decisions about the patient's care other than individuals the patient would prefer. The physician should not delay these discussions, because the significant rate of dementing disorders associated with HIV infection could result in the patient losing the competence required to complete advance directives.

Patients who complete advance directives should be encouraged to be as detailed as they prefer about what they want done or not done during the end stages of their disease. This information will be invaluable to their caregivers later by providing them specific guidance. Useful adjuncts such as the "Medical Directive"[10] and "Values History"[11] exist for this purpose. Some states restrict the use of either or both of these documents during pregnancy. Nonetheless, they should be offered, because they may be needed in the postpartum period.

### CONCLUSIONS

Appropriate clinical management of the HIV-infected pregnant woman involves multifaceted ethical challenges. We have argued that rights-based approaches, based on a civil rights model of the ethics of HIV infection, are not adequate to clinically address these challenges. We have argued, instead, for a more balanced and clinically relevant ethical framework that emphasizes the beneficence-based and autonomy-based obligations of the physician of the pregnant woman, as well as the beneficence-based obligations of the pregnant woman to the fetal patient. The ethical principle of respect for autonomy, in particular, shapes the counseling process about termination of pregnancy, contraception, and advance directives.

### REFERENCES

1. McCullough LB, Chervenak FA: Ethics in Obstetrics and Gynecology. New York: Oxford University Press, 1994.
2. Essex-Sorlie D: The Americans With Disabilities Act. I. History, summary, and key components. Acad Med 69: 519–524, 1994.
3. Americans With Disabilities Act of 1990. Public Law No. 101-336, 104 Stat 327.
4. Minkoff HL, De Hovitz JA: Care of women infected with the human immunodeficiency virus. JAMA 266: 2253–2258, 1991.
5. Kass NE: Policy, ethics, and reproductive choice: Pregnancy and childrearing among HIV-infected women. Acta Pediat Suppl 400:95–98, 1994.
6. American College of Obstetricians and Gynecologists: Human immunodeficiency virus infection: Physician's responsibilities. ACOG Committee Opinion 130. Washington, DC: American College of Obstetricians and Gynecologists, 1993.
7. Beauchamp TL, Childress JF: Principles of Biomedical Ethics. 4th ed. New York: Oxford University Press, 1994.
8. Conner EM, Sperling RS, Gelber R, Kisilev P, Scott G, O'Sullivan MJ: Reduction of maternal-infant transmission of human immunodeficiency virus type I with zidovudine treatment. N Engl J Med 331:1173–1180, 1994.
9. Volberding PA, Lagakos SW, Grimes JM, Stein DS, Rooney J, Meng TC, et al.: A comparison of immediate with deferred zidovudine therapy for asymptomatic HIV-infected adults with CD4 cell counts of 500 or more per cubic millimeter. N Engl J Med 333:401–407, 1995.
10. Emanuel L, Emanuel EJ: The medical directive: A new comprehensive advance care document. JAMA 261: 3288–3293, 1989.
11. Doukas DJ, McCullough LB: The values history: The evaluation of the patient's values and advance directives. J Fam Practice 32:145–151, 1991.
